# Development and Characterization of Conformation-Preferring Antibodies Targeting Phosphorylated Threonine 19 in PSD-95

**DOI:** 10.1523/ENEURO.0016-26.2026

**Published:** 2026-07-02

**Authors:** Jacob Alvarez, Prajwal Kurup, Lauren Kholsaat, Sai Kanuru, Esmeralda Paredes, Het Gor, Aliza J. Bernardo, Bota Tulegenova, Kaetlyn S. Khoury, Micaela Herrera, Pedro Castrejon, Jary Y. Delgado

**Affiliations:** Department of Biology, Loyola University Chicago, Chicago, Illinois 60660

**Keywords:** *cis*–*trans*, isomerization, LTD, phosphorylation, proline-directed kinases, PSD-95

## Abstract

Activity-dependent synaptic plasticity is governed by posttranslational mechanisms that regulate the stability and molecular organization of postsynaptic protein complexes. Proline-directed phosphorylation of the N-terminus of PSD-95 promotes synaptic weakening during NMDAR-dependent LTD, yet this type of phosphorylation also alters the *cis*–*trans* isomerization of the adjacent peptidyl-prolyl bond. Despite its predicted importance, these conformational changes have not been directly measured using existing molecular tools. Here we describe the development of novel conformation-preferential antibodies that distinguish structural states of PSD-95 when threonine 19 (T19), a site implicated in NMDAR-LTD, is phosphorylated. These antibodies were validated biochemically and in cellular assays, where signal increased following GSK3β-mediated phosphorylation and was lost upon dephosphorylation. These reagents represent the first conformation-preferential antibody-based tools capable of reporting phosphorylation-dependent conformational states of PSD-95 at T19. This strategy validates and expands prior framework for developing conformational-sensitive antibodies, an approach that can be applied to other synaptic proteins.

## Significance Statement

Posttranslational modifications regulate synaptic proteins not only through changes in chemical composition but also by altering protein conformation. Phosphorylation of PSD-95 at threonine 19 is required for NMDA receptor–dependent synaptic weakening, yet the associated *cis*–*trans* isomerization of the adjacent proline bond has been inaccessible to experimental analysis. In this study, we introduce the first conformation-preferential antibodies capable of distinguishing phosphorylation-dependent structural states of PSD-95 at this site. These reagents provide a new molecular toolkit for investigating how proline-directed phosphorylation and isomerization regulate synaptic scaffolds during plasticity and disease.

## Introduction

Activity-dependent change in synaptic strength is the leading proposed cellular mechanism responsible for learning and memory (Malenka and Bear, 2004); however, when these processes are dysregulated, there can be an exaggerated loss of synapses. In particular, the process of *N*-methyl-d-aspartate receptors (NMDAR)–dependent long-term depression (NMDAR-LTD) has been associated with the loss of synapses that is associated with Alzheimer's disease, bipolar disorder, and schizophrenia (Howes et al., 2024). A key molecular player participating in the expression of NMDAR-LTD is postsynaptic density protein 95 (PSD-95; Migaud et al., 1998; Ehrlich et al., 2007; Carlisle et al., 2008; Xu et al., 2008; Dore et al., 2021).

PSD-95 is extremely abundant at the synapse (Chen et al., 2011; Alkaas et al., 2025). PSD-95 is a multidomain protein composed of three PDZ binding domains, an SH3 domain, and a nonfunctional guanylyl kinase domain (Woods and Bryant, 1993). In addition to the structured domains, PSD-95 contains two unstructured regions, one is located at the N-terminus domain of the protein, and the other unstructured domain is located between the second and third PDZ domains. The unstructured regions in PSD-95, as well as in many other proteins, are thought to determine the fate of the protein (Vallejo et al., 2017; Alkaas et al., 2025). For instance, these regions are subjected to posttranslational modifications which determine the fate of the protein. The N-terminus domain of PSD-95 is subjected to a multitude of posttranslational modifications, some of these determine the synaptic enrichment of PSD-95 at synapses and whether the protein is removed during the process of NMDAR-LTD (Topinka and Bredt, 1998; Morabito et al., 2004; Ho et al., 2011; Nelson et al., 2013). During LTD, the N-terminus domain of PSD-95 is subjected to phosphorylation at threonine 19 (T19) and possibly at serine 25 (S25; Morabito et al., 2004; Nelson et al., 2013). The phosphorylation of these two sites destabilizes PSD-95 post the induction of LTD, which allows the internalization of the α-amino-3-hydroxy-5-methyl-4-isoxazole propionic acid (AMPA) receptor complex, highlighting PSD-95's crucial role in regulating synaptic plasticity and function in response to NMDAR-LTD. These same phosphorylation sites, residing within the N-terminus PEST domain, were shown to alter the conformational state of this region of PSD-95 (Delgado, 2020). The observed conformational change in this region is likely the result of *cis-to-trans* isomerization of the peptidyl-prolyl bonds present within the N-terminus domain.

*Cis–trans* isomerization of peptidyl-prolyl bonds is an essential physiological process regulating almost every aspect of cellular physiology (Yaffe et al., 1997; Ryo et al., 2003; Yeh and Means, 2007; Zhou and Lu, 2016). *Cis–trans* isomerization refers to the 180° half rotation around the peptidyl bond in prolines (Lu et al., 2007). This conformational change can trigger large or subtle, local, changes in the conformation of the protein. This molecular mechanism is unique to prolines as their peptide bond can exist in two isomers (*cis* or *trans*). In nature, the *trans* isomer is the most abundant with some proteins experiencing up to 95% of a particular proline in the *trans* isomer; however, in some cases the *cis* isomer is the more prevalent. This equilibrium can be altered when a serine or a threonine (preceding a proline) becomes phosphorylated by a proline-directed kinase. Therefore, this mechanism occurs on both phosphorylated and nonphosphorylated peptidyl-prolyl bonds. Phosphorylation just alters the abundance of a certain species.

Besides the well-characterized effects of a few proline-directed serine/threonine (S/T-P) kinases, it is not well understood how the change in *cis–trans* equilibrium after phosphorylation alters the biological significance of the phosphorylated protein. What is better understood is the role of the peptidyl-prolyl isomerases (Pin1), which is the only phosphorylation-dependent *cis–trans* isomerase in our genome (Holzer et al., 2002; Pastorino et al., 2006; Nakamura et al., 2012; Park et al., 2013). For example, knockouts of Pin1 show signs of age-related neuronal damage (Pastorino et al., 2006). At inhibitory synapses, Pin1 associates with phospho-gephyrin to promote binding to glycine receptors (Moretto-Zita et al., 2010) and with phosphorylated neuroligin2 (NL2) to block NL2 binding with gephyrin (Antonelli et al., 2014). At excitatory synapses of medium spiny neurons, Pin1 mediates the *cis–trans* isomerization of the metabotropic glutamate receptor 5 (mGluR5), a cellular mechanism implicated in cocaine sensitization (Park et al., 2013). In the hippocampus, Pin1 was shown to negatively regulate postsynaptic dendritic spines and the amount of *N*-methyl-d-aspartic acid receptors (NMDAR) at synapses (Antonelli et al., 2016). Our recently published findings extend the previous findings on the effects of Pin1 at excitatory synapses by demonstrating molecular mechanism linking Pin1 action to NMDAR stability (Delgado et al., 2020). In our work, we provide evidence for Pin1 association with phosphorylated T19 and S25 in the N-terminus domain of PSD-95. We did show that Pin1 association blocks palmitoylation of C3 and C5, a posttranslational modification necessary for PSD-95 synaptic stabilization (Topinka and Bredt, 1998). The reduction in PSD-95 palmitoylation correlated with a decreased amount of PSD-95 in postsynaptic dendrites and spines and fewer functional excitatory synapses. Lastly, we demonstrated that this association induced a conformational change in PSD-95, attributable to the phosphorylated residues in the N-terminus domain of the protein.

These examples provide evidence supporting the importance of both proline-directed phosphorylation and Pin1 action; however, this previously published work did not describe the precise change in *cis*/*trans* equilibrium induced by proline-directed phosphorylation (Delgado, 2020). More importantly, we do not know if the phosphorylation of T19 in PSD-95 results in a change in *cis–trans* equilibrium at phospho-T19, a molecular event that may play a key role in the induction or duration, or expression of NMDAR-LTD (Chowdhury and Hell, 2019). In this paper we tackle this problem by describing the methods utilized for the development of conformational preferring antibodies capable of distinguishing between *cis* and *trans* isomers of phosphorylated T19 in PSD-95.

## Materials and Methods

### Peptide synthesis

Peptide synthesis was carried out in the Peptide Synthesis Core at Northwestern University using automated solid phase peptide synthesis. Non-natural amino acid couplings were carried out on a mechanical shaker after manual addition of the components. This allowed for more control of the reaction equivalents and conditions. Phosphorylation of T19 was introduced during synthesis where indicated. Hydroxyproline and ʟ-5,5-dimethylproline (DMP), as proline analogs, were added to the peptides on resin using standard amide bond forming coupling reagents. All peptides were purified by reversed phase HPLC to 95% purity or greater, lyophilized to dryness, and characterized by LCMS. Predicted *cis*–*trans* ratios were assigned based on prior biophysical characterization of proline analogs (Nakamura et al., 2012).

Synthetic peptides corresponding to the PSD-95 N-terminal region encompassing T19 were designed to evaluate the contribution of proline isomerization to antibody recognition. All peptides shared a common backbone sequence (CKYRYQDEDTPPLEHSP), with modifications introduced at the T19–Pro20 motif as indicated in Table 1. An N-terminal cysteine residue was included to facilitate peptide conjugation.

**Table 1.** Synthetic peptides used for antibody generation and characterization

Peptide	Short name	Sequence	*Cis*/*trans* ratio	Use
(1) phospho-T19-proline	pT19P	C-Ahx-YRYQDEDpTPPLEH-amide	0.1	Step #3 purification/competition assays
(2) phospho-T19-homoproline	pT19-Pip	C-Ahx-YRYQDEDpT(Pip)PLEH-amide	3.1	Rabbit inoculation
(3) *cis*-lock-in phospho-5,5-dimethyl-ʟ-proline	pT19-DMP (cis-lock)	C-Ahx-YRYQDEDpT(Dmp)PLEH-amide	24	Steps #1 and #4 purification
(4) *trans*-lock-in phospho-T19-alanine	pT19-Ala (*trans*-lock)	C-Ahx-YRYQDEDpTAPLEH-amide	0	Step #2 purification (left column)
(5) alanine-19-proline	AP	C-Ahx-YRYQDEDAPPLEH-amide		competition assays

To bias the conformational state of the peptide backbone, proline analogs with defined *cis*–*trans* preferences were incorporated at position Pro20. In addition to the native T19–Pro sequence, phosphorylated variants were generated containing either natural proline, pipecolic acid (Pip), 2,2-dimethylproline (Dmp), or alanine substitutions. Pipecolic acid (also known as homoproline) and 2,2-dimethylproline were selected based on their established ability to stabilize the *cis* peptide bond conformation, whereas alanine substitution was used to favor a *trans* configuration.

### Antibody production, purification, and screening

Custom polyclonal rabbit antibodies were generated by YenZym Antibodies using modified peptide antigens derived from human DLG4 (PSD-95) residues 12–24 [sequence: C-Ahx-YRYQDEDT(Pip)PLEH-amide]. Pip (homoproline) was used as it significantly increases the content of peptides in *cis* to up to 70% (Nakamura et al., 2012). The modified peptide was synthesized and conjugated to keyhole limpet hemocyanin (KLH) to enhance immunogenicity. For the immunizations, 5 mg of peptide–KLH conjugate was used to immunize two rabbits under a 66 d immunization regimen consisting of five injections (Days 0, 14, 28, 42, and 56). Pre-immune (5 ml) and test-bleed (5 ml) sera were collected from each rabbit to assess specificity by ELISA, and ∼50 ml of production antiserum was obtained at Day 66.

**Figure 1.**
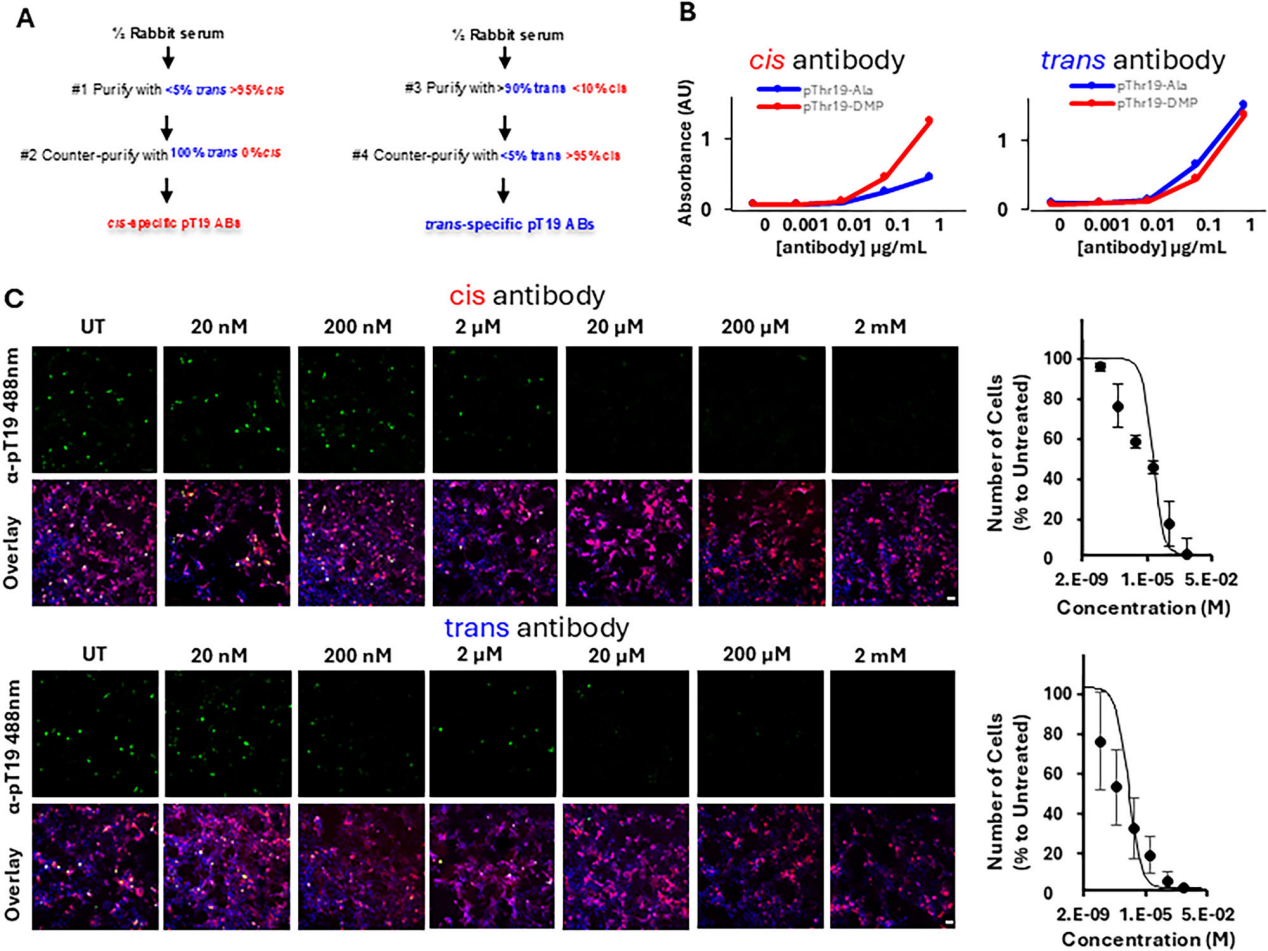
Development and validation of conformation-preferring antibodies targeting the *cis* and *trans* phosphorylated states of PSD-95 at T19. ***A***, Schematic representation of the antibody generation process, showing purification strategies for isolating *cis*- and *trans*-preferring antibodies. The figure indicates the step at which each peptide was used, and the corresponding sequences are provided in Table 1. ***B***, ELISA binding curves demonstrating the specificity of *cis*- and *trans*-antibodies for their respective conformations. ***C***, Immunofluorescence imaging of pT19 at varying doses of the phosphorylated T19 peptide and nonphosphorylatable peptide (Extended Data Fig. 1-1*B*). HEK293T cells coexpressing PSD-95 and GSK3β-RFP labeled with cis (top) or trans (bottom) antibodies, anti-PSD-95, and anti-HA. Scale bar: 50 µM. Cells were stained with DAPI (blue), *cis* or *trans* pT19 (green), and anti-PSD-95 (magenta). For all peptide concentrations (shown above; UT, untreated), the *cis* or *trans* and the overlay images are shown. Quantification of cell number per field of view normalized to no peptide control. Right, A sigmoidal dose–response curve was fitted to the data. The response of the antibody to dephosphorylation is tested in Extended Data Figure 1-1*A*.

For ELISA assays, ∼1 mg of each peptide was used. For affinity purification, 4 mg of each unconjugated peptide containing the N-terminal Cys-Ahx linker was immobilized via the cysteine residue onto activated agarose resin (Thermo Scientific, gravity-flow column format). Primary affinity purification was performed by passing clarified antisera separately from each rabbit over columns coupled to the various peptides, yielding antibody fractions enriched in the *cis* or *trans* isomer. The eluates were subsequently affinity-absorbed against the opposite isomer to deplete any possible cross-reactive antibodies, yielding highly enriched fractions (Fig. 1*A*). The second flow-through contained either the *cis*- or *trans*-conformation preferred antibodies, while the bound fraction represented cross-reactive antibody recognizing both modified and non-modified forms (this fraction recognizes total PSD-95). Both populations were recovered, dialyzed into phosphate-buffered saline (PBS), pH 7.4, containing 50% glycerol, aliquoted, and stored at −20°C.

Antibody specificity was evaluated by indirect ELISA using plates coated with either modified or non-modified peptides. The relative binding profiles were compared across pre-immune, test-bleed, and affinity-purified antibody samples to confirm specificity toward the modified antigen.

### HEK 293T cell culture

HEK 293T cells were plated in DMEM media supplemented with 10% FBS and 1× Pen/Strep as described in our previous publication (Delgado, 2020). Cells were plated onto 100 mm plates at confluency of 10,000,000 cells per plate. Four to six hours post-plating, cells were then transfected using the calcium phosphate transfection method with plasmids expressing PSD-95 (wt), GSK3β:RFP, or HA-GSK3β. Approximately 3 µg of each cDNA was used per plate for each transfection. Cells were then placed back in the incubator and left to grow. Cells were harvested 48 h posttransfection by scraping them off in 1× PBS at 4°C. Samples were then spun at 800 × *g* for 5 min at 4°C in a tabletop centrifuge to separate the cell pellet. The resulting supernatant was aspirated, and the pellets were immediately frozen at −80°C.

### SDS-PAGE electrophoresis and Western blotting

Protein samples were prepared from the frozen pellets. Cells were lysed in a buffer containing 50 mM Tris-HCl, 200 mM NaCl, 100 mM NaF, 10% glycerol, 1% Triton X-100, and protease inhibitor cocktail III (Calbiochem) to pH 8. The nuclear and mitochondrial fraction was removed by centrifugation at 800 × *g* for 10 min at 4°C. The resulting pellet was sonicated briefly (for 3 s at 4°C) on an ultrasonic homogenizer and the resuspended solution is spun once more. The resulting supernatant was collected, and the protein concentration quantified using the BCA method.

For regular Western blots, equal amounts of protein were mixed with 4× Laemmli buffer, diluted to 1×, and boiled at 95°C for 6 min. Proteins were separated on 10% SDS-polyacrylamide gels at 120 V for ∼90 min in running buffer (25 mM Tris, 19 mM glycine, 0.1% SDS). Following electrophoresis, the proteins were transferred to PVDF membranes using a wet transfer system at 100 V for 1 h on ice (Bio-Rad Mini Trans-Blot Cell and Criterion Blotter). The membranes were blocked in 1% BSA in TBST (Tris-buffered saline with 0.1% Tween 20) for 1 h at room temperature. Primary antibodies against PSD-95 (K28/43, at a dilution of 1:400, from cell supernatant) and phospho-T19 (YZ8553, at a dilution of 1:5,000) were diluted in 4% nonfat milk in TBST and incubated overnight at 4°C in a rocker. For the phospho-T19 experiment, membranes were blocked in 4% BSA. The next day, membranes were washed three times in TBST and incubated in 4% nonfat milk in TBST with SouthernBiotech HRP-conjugated secondary antibodies at a 1:10,000 dilution for 2 h at room temperature. Protein bands were visualized using enhanced chemiluminescence (ECL) and detected using a chemiluminescence imaging system (Odyssey XF Imaging System, LICORbio). Images were imported to FIJI, and the intensity of the protein bands was quantified using the gel analyzer function. The ratios of ubiquitinated PSD-95 to total PSD-95 were calculated for statistical comparison.

### Dephosphorylation assay

HEK293T cells were transfected with PSD-95 and lysed as described before. Between 200 µg of total protein (depending on cell confluence) were incubated with 2 µg of anti-PSD-95 antibody (K28/43) for a minimum of 1 h followed by 2 h incubation with 15 µl of Cytiva Protein G Mag Sepharose (Fisher Scientific™) pre-blocked with 1% BSA. In magnetic rack, wash beads three times with lysis buffer, transferring to a new Eppendorf low binding tube for the third wash. The lysates were split into two. The sample receiving the λ-phosphatase was resuspended in 50 µl of NEBuffer (39 µl of water, 5 µl of 10× NEBuffer pack for protein MetalloPhosphatases, 5 µl of MnCl_2_, and 1 µl of λ-protein phosphatase). The negative control remained in 50 µl of lysis buffer. Both samples were incubated at room temperature for 45 min while rocking followed by the addition of 4× Laemmli buffer (with DTT), boiling for 5 min, and centrifugation to collect any condensation. Beads were removed using the magnetic rack. Samples were then run in a 12% SDS-PAGE gel.

### Immunocytochemistry

HEK 293T cells were transfected as explained previously. Forty-eight hours post transfection, HEK 293T cells were fixed in 4% paraformaldehyde at room temperature for 10 min. Cells were then rinsed three times with 1× PBS, then 5 min in 50 mM NH_4_Cl, and three more quick rinses in 1× PBS. Cells were permeabilized in 0.1% Tx-100 PBS for 10 min followed by three quick PBS rinses. Cells were incubated in freshly made 0.5% sodium borohydride in PBS for 5 min. Cells were rinsed thoroughly in PBS and incubated in 1 ml of 1% BSA in PBS for 45 min followed by incubation in 100 µl of *cis* or *trans* anti-pT19 (1:500, or 0.05 µg total) and anti-PSD-95 (1:1,000) for 1 h or overnight at 4°C (Table 1). For peptide competition experiments, antibodies were preincubated with the phosphorylated peptide prior to immunohistochemistry, and the antibody–peptide mixture was then applied to cells. Cells were rinsed three times in PBS and Alexa Fluor 647 anti-mouse (1:500) and Alexa Fluor 488 anti-rabbit applied at a dilution of 1:500 for 2 h in 1% BSA in PBS. Cells were rinsed five times in PBS, postfixed in 4% PFA and mounted in Mowiol.

### Confocal imaging

Confocal imaging was performed using a Zeiss LSM 880 Airyscan confocal system. In brief, neurons were imaged in non-modified Hibernate-E media. Transfected neuron localization was facilitated through visual guidance, employing an Illuminator HXP 120 V Halogen lamp alongside a 10× apo (0.45 NA) M27 objective, coupled with an EGFP emission filter (BP 420-480 for excitation and BP 495-550 for emission). Once the cells were localized, the objective was changed to a Plan-Apochromat 63×/1.40 Oil DIC f/ELYRA objective. Images were acquired using the solid state 488 nm laser to excite EGFP and engaging the Airyscan module. Laser power (2–5% laser power) was adjusted to maximize signal and avoid photobleaching/photodamage. *Z*-stacks were collected via the control of a completely motorized stand equipped with definite focus 2 stabilization unit.

### Experimental design and statistical

Data collection was interleaved, controlling time and order effects. Sample size was determined using a power analysis calculator based on the known variability of a sample experiment. For each experiment, data from all groups were acquired weekly to reduce variability or else it was not included in the analysis. For biochemistry, unpaired and one-way ANOVAs were utilized. Normality testing was performed on every group using D'Agostino and Pearson’s omnibus normality test. Between-group statistical significance was calculated accordingly for each distribution and experiment design. Data were normalized on a weekly basis to compensate for week-to-week variability. Numerical averages are presented as mean ± SEM or as box plots. Statistical analyses were created using Excel or using R. Exact *p* values are reported when provided. At the end of a dataset, the groups were tested for the presence of outliers using the Prism online calculator at a significance level of *p* < 0.05. Only then were these eliminated.

Dose–response data were analyzed by fitting to a four-parameter Hill equation of the form:

$$y=\mathrm{Bottom}+\frac{\mathrm{Top}\text{–}\mathrm{Bottom}}{1+(X/EC_{50})},$$

where *y* represents the normalized response, *x* is the peptide concentration, Top and Bottom correspond to the asymptotic maximum and minimum responses, EC_50_ is the concentration producing half-maximal response, and *n* is the Hill coefficient.

EC_50_ and Hill coefficients were estimated by fitting dose–response data to a four-parameter Hill equation using nonlinear least-squares regression with Levenberg–Marquardt optimization to minimize the residual sum of squares between observed and predicted values. Data are presented as mean ± SEM.

## Results

The N-terminus domain of PSD-95 undergoes conformational changes in response to proline-directed phosphorylation of T19 and S25, and these sites are targets of the proline isomerase Pin1 (Delgado, 2020). Pin1 binding to these residues can accelerate the rate of *cis–trans* isomerization, in a phosphorylation-dependent manner. Furthermore, the mutation of these sites to alanine alters the conformation of PSD-95, leaving PSD-95 in a more extended open conformation. While these findings are informative, the results do not allow us to understand the dynamics of *cis*-to-*trans* isomerization of phosphorylated T19 or S25 in cells. This type of dynamics is often studied using NMR of peptides in solution; however, very few examples show the abundance of these isoforms of a phosphorylated epitope in vivo, except for phosphorylated threonine 231 in tau (Nakamura et al., 2012).

Therefore, we generated a polyclonal antibody in rabbits designed against the phosphorylated T19 in the N-terminus domain of PSD-95, to enable conformation-preferential detection of these phosphorylated states in cells. The serum of the rabbit was first screened for a positive immune response before a second round of antibody boosting. The serum was then extracted and purified following the screening diagram shown in Figure 1*A*. Half of the serum was used to generate *cis* conformation-preferring antibodies, and the other half was used to select for *trans*-conformation-preferring antibodies. The *cis*-specific antibodies were first purified against a column coupled with the *cis*-locked in peptide *cis*-lock-in phospho-5,5-dimethyl-ʟ-proline, predicted to be 95% of the time in the *cis* isomer Table 1 (Nakamura et al., 2012). The bound antibodies were eluted and then counter purified using the *trans*-lock-in phospho-T19-alanine, predicted to be 100 percent of the time in the *trans* isomer. This strategy yielded our *cis*-preferred phosopho-T19 antibodies. The *trans* antibodies were first purified against a column coupled with the *trans* peptide phospho-T19-proline, predicted to be over 90 percent of the time in the *trans* isomer Table 1. The bound antibodies were eluted and then counter purified using the *cis*-lock-in phospho-5,5-dimethyl-ʟ-proline, predicted to be over 95% of the time in the *cis* isomer. This strategy yielded our *trans*-preferred phospho-T19 antibodies.

10.1523/ENEURO.0016-26.2026.f1-1 Figure 1-1 Antibodies demonstrate selectivity towards the phosphorylated versions of T19. (A) Western blot analysis showing phosphatase sensitivity and preference of the *cis*-pT19P and *trans*-pT19P antibodies. Treatment with λ-phosphatase resulted in decreased levels of phosphorylation detected by both the *cis*-pT19 (left) and *trans*-pT19 (right) antibodies. (B) Immunofluorescence imaging of pT19 at varying doses of non-phosphorylated peptide. HEK293 T cells co-expressing PSD-95 and HA-GSK3β labeled with conformation-specific antibodies, anti-PSD-95, and anti-HA. Scale bar: 50 µM. Quantification of cell number per field of view normalized to untreated condition (UT) and fitted to a sigmoidal dose-response curve presented to the right. Download Figure 1-1, TIF file.

Having obtained the antibodies, we determined the specificity of the antibodies in an ELISA assay. The *cis*- and *trans*-specific antibodies were challenged with wells coated with the *cis*- or *trans*-locked-in peptides. While the purified *cis*-specific fraction selectively recognized the *cis*-locked in peptides (1.26 vs 0.474 absorbance units), the *trans*-specific fraction failed to demonstrate any selectivity for either isomer (1.52 vs 1.38; Fig. 1*B*). Thus, suggesting that our purification strategy is more effective at generating antibodies able to recognize the *cis* isomer.

Following the direct ELISA binding curve experiment that verified *cis–trans* conformational specificity, next we tested if the anti-pT19 antibodies are phosphorylation specific and work in immunocytochemistry experiments. To do this, we stain HEK 293T cells expressing PSD-95 with the *cis*-pT19 or *trans*-pT19 antibody. The specificity of the antibodies against the phosphorylated form of the T19 epitope was tested doing a titration competition experiment using a phosphorylated T19 peptide (Table 1, peptide #1). Cells were then imaged in the confocal microscope and the intensity of the signal quantified (Fig. 1*C*). The staining intensity and number of positively stained cells remained largely stable across a broad range of phospho-peptide concentrations (shown above each image), with a reduction apparent at relatively low concentrations of the peptide (Fig. 1*C*; cis: UT 100.00 ± 7.95; 20 nM 95.75 ± 11.33; 200 nM 75.68 ± 3.31, 2 µM 57.43 ± 3.19, 20 µM 44.79 ± 11.06, 200 µM 16.22 ± 1.89, 2 mM 0.77 ± 0.47; trans: UT 100.00 ± 32.11, 20 nM 73.50 ± 24.06, 200 nM 50.75 ± 18.64, 2 µM 30.63 ± 14.93, 20 µM 17.50 ± 9.18, 200 µM 5.00 ± 4.05, 2 mM 1.25 ± 1.25; *n* = 5). Sigmoidal curve fitting revealed low EC_50_ values for competitive inhibition by the phosphorylated peptide for both the *cis* and *trans* antibody samples (*cis* EC_50_ ≈ 20 µM; Hill Coefficient *n* ≈1.4–1.7, and the *trans* EC_50_ ≈ 1 µM; Hill coefficient *n* ≈ 1.1–1.3). Taken together, the images demonstrate that phospho-dependent competition occurs for both the *cis* and *trans* antibodies, suggesting that both fractions of *cis*- and *trans*-pT19 antibodies are phospho-specific. This data indicates that the purification procedure, although not specifically selective for a phosphorylated antibody, purified *cis*- and *trans* antibodies that recognized phosphorylated T19, as describe previously (Nakamura et al., 2012).

After confirming pT19 antibody signal was blocked by peptide competition, we further tested its phosphorylation specificity. Specifically, we tested for a signal decrease in response to dephosphorylation under biochemical conditions. To do this, we performed a complementary lambda phosphatase (λ-phosphatase) treatment assay (Extended Data Fig. 1-1*A*). Immunoprecipitated PSD-95 from cell lysates were incubated with a broad-spectrum protein phosphatase (λ-phosphatase). Samples were then eluted and ran in a Western blot. Western blot analysis revealed that λ-phosphatase treatment resulted in a substantial reduction in signal for both *cis*-pT19 and *trans*-pT19 antibodies. Treatment of the sample probed with the *cis* antibodies resulted in a 51% reduction, and the sample probed with the *trans* antibodies resulted in a 21% reduction in signal [Extended Data Fig. 1-1*A*, cis (+) λ-phosphatase 0.49 ± 0.06, *p* < 0.05, 5 repetitions; trans (+) λ-phosphatase 0.79 ± 0.26, *p* = 0.810, 4 repetitions]. Notably, dephosphorylation had a more pronounced effect on the *cis*-pT19 signal relative to *trans*-pT19, replicating the differential sensitivity of these antibody fractions observed in the ELISA experiment. Also, we further assess the specificity of the antibodies against the nonphosphorylated form of the T19 epitope using the titration competition experiment against the nonphosphorylated T19 peptide (Extended Data Fig. 1-1*B*). For both the *cis* and *trans* antibodies, the number of positively stained cells remained largely stable across a broad range of nonphospho peptide concentrations, with a reduction apparent only at the highest concentrations tested (Extended Data Fig. 1-1*B*; *cis*: UT 100.00 ± 26.88; 20 nM 100.00 ± 10.69; 200 nM 87.78 ± 9.36; 2 µM 87.78 ± 12.60; 20 µM 94.44 ± 13.83; 200 µM 57.78 ± 14.23; 2 mM 6.67 ± 2.72. *trans*: UT 100.00 ± 16.94; 20 nM 74.19 ± 10.62; 200 nM 106.50 ± 13.35; 2 µM 100.00 ± 11.89; 20 µM 100.81 ± 8.89; 200 µM 63.41 ± 8.35; 2 mM 17.89 ± 1.89; *n* = 5). Sigmoidal curve fitting revealed high EC_50_ values for competitive inhibition by the nonphosphorylated peptide for both the cis antibody (EC_50_ ≈ 200 µM; Hill coefficient *n* ≈ 5–7) and the trans antibody (EC_50_ ≈ 150 µM; Hill coefficient *n* ≈ 2.5–3.5). These values contrast the EC_50_ values obtained from the phospho-peptide competition, where inhibition occurred at substantially lower concentrations for both the antibodies. These results are consistent with the phospho-dependent specificity established by the phospho-peptide competition assay (Fig. 1*C*). Together with the peptide competition assay, these results establish that the antibodies are phospho-dependent and provide complementary validation through both cellular and biochemical approaches.

**Figure 2.**
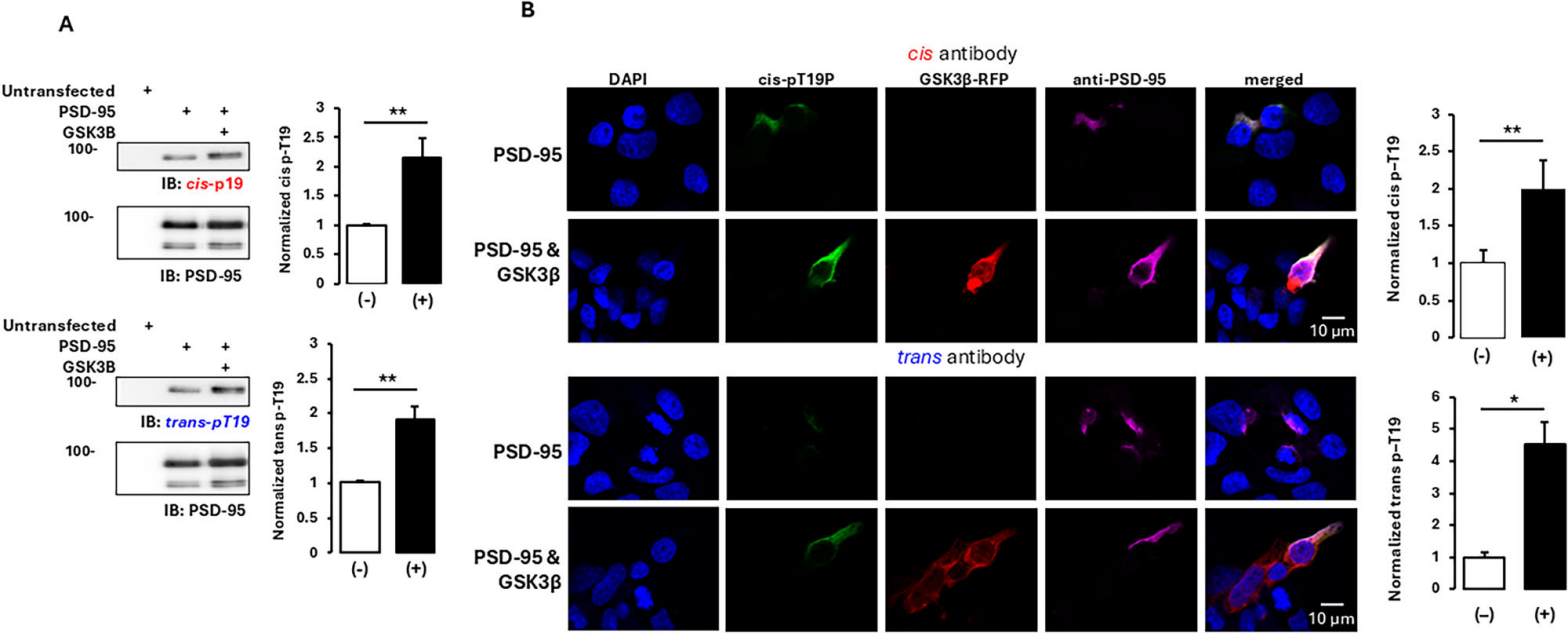
The *cis* and *trans* antibodies are sensitive to manipulations that increase T19 phosphorylation. ***A***, Western blot analysis of HEK293T cells expressing PSD-95 and GSK3β showing an increase in *cis*-pT19P (top) and *trans* (bottom) T19 signal in response to GSK3β overexpression. Quantification of the signal indicate an increase in the levels of phosphorylation signal detected by both the *cis*-pT19 (left) and *trans*-pT19 (right) antibodies in the presence of GSK3β. The response of the antibody to the T19A mutant is shown in Extended Data Figure 2-1. ***B***, Pseudocolor images of HEK293T cells overexpressing PSD-95 alone (top row) or with GSK3β-RFP (bottom row). Cells were stained with DAPI (blue), anti-*cis*-pT19 (green), and anti-PSD-95 (magenta). Final box shows the merged image. ***B***, The bottom two rows shows HEK 293T cells transfected and stained with identical conditions to the top row but stained with the *trans*-pT19 antibody.

Next, we sought to confirm that our antibodies could recognize increases in phosphorylation. Previous research has demonstrated an increase in the phosphorylation of T19 on PSD-95 when GSK3β was overexpressed (Nelson et al., 2013). To verify that our antibodies can also detect the increase in T19 phosphorylation when GSK3β is overexpressed, HEK 293T cells were transfected with plasmids expressing PSD-95 and GSK3β. Cell lysates were run in a Western blot and stained using the *cis* and *trans* antibodies. For both antibodies, there was close to a twofold increase in the condition where GSK3β was overexpressed specifically [Fig. 2*A*; cis, (−) GSK3β 1.02 ± 0.02, (+) GSK3β 2.20 ± 0.34, *n* = 9, *t* test, *p* < 0.005; trans, (−) GSK3β 1.02 ± 0.01, (+) GSK3β 1.91 ± 0.20, *n* = 5, *t* test *p* = 0.008]. Notably, there is a slightly larger increase in the phosphorylated *cis*-T19 than there is an increase of the phosphorylated *trans*-T19. While our previous findings demonstrated a bias toward the signal recognized by the *cis* antibody fraction, this data suggests that the increase in phosphorylation leads to an increase in signal detected by both species of the antibodies.

10.1523/ENEURO.0016-26.2026.f2-1 Figure 2-1 The cis-pT19 antibody displays higher preference towards the phosphorylatable form of PSD-95 at T19. Western blot images generated using the cis-pT19 (A) and trans-pT19 (B) antibodies from samples transfected with WT PSD-95 or PSD-95 T19A and HA-GSK3β. Graphs show quantification of normalized pT19 phosphorylation as a fraction of total PSD-95. Error bars represent SEM. Download Figure 2-1, TIF file.

To validate the biochemical results, HEK 293T cells were cotransfected with PSD-95 and GSK3β-RFP and the cells were immunostained. While signal is detectable in the no GSK3β condition, there was a significant increase in signal observed in the presence of GSK3β for both (Fig. 2*B*; *cis*: no GSK3β 1 ± 0.17, *n* = 10; with GSK3β 1.99 ± 0.40, *n* = 14, *t* test, *p* = 0.04; and *trans*: no GSK3β 1 ± 0.16, *n* = 21; with GSK3β 4.53 ± 0.67, *n* = 24, *t* test, *p* < 0.001), which matches with the increased in phosphorylation observed in the biochemical experiment (Fig. 2*A*).

To further confirm T19 phospho-specificity, we repeated this experiment using a phospho-dead T19A mutant of PSD-95, in which the threonine at position 19 is replaced by alanine and therefore cannot be phosphorylated (Extended Data Fig. 2-1). In cells expressing wild-type PSD-95, GSK3β overexpression produced the expected increase in signal for both the *cis* and *trans* antibodies, consistent with Figure 2*A* (Extended Data Fig. 2-1; one-way ANOVA with Tukey HSD post hoc correction; cis: *F*_(2,15)_ = 24.35, *p* < 0.001; trans: *F*_(2,15)_ = 7.76. *n* = 6). In cells expressing the T19A mutant, a trend was observed; however, this change in signal was not statistically significant, possibly due to the dataset being underpowered (Extended Data Fig. 2-1). Because alanine cannot be phosphorylated, this residual T19A signal likely reflects low-level cross-reactivity of the polyclonal antibody preparations with the surrounding nonphosphorylated epitope, a recognized property of phosphorylation state-specific polyclonal antibodies (Czernik et al., 1991). Collectively, we have developed the first conformation and phosphorylation-specific antibodies against phosphorylated T19, providing a tool for studies aiming at characterizing the role of PSD-95 in synaptic plasticity and synaptic transmission.

## Discussion

In this study, we report the development of phosphorylation-dependent, conformation-selective antibodies against the Thr19–Pro motif of PSD-95. This work builds upon pioneering efforts by Nakamura et al. (2012), who developed conformation-specific antibodies against phosphorylated Thr231 in tau, a site later shown to serve as a biomarker for Alzheimer's disease pathology (Suárez-Calvet, 2022). Extending this strategy to a core synaptic scaffold protein, we generated antibodies capable of distinguishing distinct structural states of phosphorylated Thr19 in PSD-95. These antibodies function across multiple biochemical and cellular applications, including ELISA, Western blotting, and immunohistochemistry.

A key outcome of this work is the availability of reagents that enable discrimination between conformational states of phosphorylated PSD-95 at Thr19. These tools create the opportunity to examine how proline-directed phosphorylation and isomerization contribute to PSD-95 regulation under basal conditions and during activity-dependent plasticity. Thr19 is an abundant phosphorylation site within the postsynaptic density of adult mouse brain, and its involvement in synaptic plasticity has been established (Coba et al., 2009; Nelson et al., 2013). The ability to distinguish conformational states at this site provides a new means to interrogate how these posttranslational modifications participate in synaptic signaling.

While our data demonstrate clear differential recognition of phosphorylated PSD-95 by the *cis*- and *trans*-preferring antibodies, interpretation of relative signal levels must be approached with caution. In immunohistochemical experiments (Fig. 2*B*), the *trans*-reactive antibody displayed a larger apparent increase in signal compared with the *cis*-reactive antibody following phosphorylation. However, this difference likely reflects technical properties of the antibodies rather than true differences in isomer abundance. Baseline levels of the *trans*-reactive signal were low, whereas the *cis*-reactive antibody produced stronger signals under both conditions. Consistent with this, the *cis*-reactive antibody required lower concentrations for detection in Western blot assays, suggesting higher apparent affinity. Because affinity constants were not directly measured, we cannot infer biological shifts in isomer abundance from signal intensity alone.

The T19A phospho-mutant data (Supplementary Fig. 2) provide additional consideration. The residual signal observed with both antibody fractions in T19A-expressing cells, and its modest elevation upon GSK3β co-expression, most likely reflects minor cross-reactivity with the nonphosphorylated epitope surrounding T19. This is consistent with a well-characterized limitation of polyclonal phosphorylation state-specific antibodies, which have been observed to retain low-level affinity for the unmodified peptide sequence they originate from (Czernik et al., 1991).

Thus, these reagents should be viewed as conformation-preferential reporters, rather than quantitative measures of *cis*–*trans* equilibrium. Nonetheless, their ability to distinguish structural states of phosphorylated PSD-95 enables a range of mechanistic experiments that were previously inaccessible, including analyses of how kinase and isomerase activity regulate PSD-95 conformation in cells and tissue.

Overall, we have developed the first conformation-preferential antibodies targeting a phosphorylated site in PSD-95. The compatibility of these reagents with both biochemical assays and immunohistochemistry provide a powerful platform for studying the spatial and activity-dependent regulation of PSD-95 conformation in synapses, and this approach should be broadly applicable to other proline-directed phosphorylation sites in neuronal proteins.

## Synthesis

Reviewing Editor: Jean Christophe Poncer, Institut du Cerveau Decisions are customarily a result of the Reviewing Editor and the peer reviewers coming together and discussing their recommendations until a consensus is reached. When revisions are invited, a fact-based synthesis statement explaining their decision and outlining what is needed to prepare a revision will be listed below. The following reviewer(s) agreed to reveal their identity: NONE.

The authors have satisfactorily addressed all the points raised by the reviewers in the previous version of their manuscript. Only one correction remains necessary: in Figures 1B and 1-1B, the scale bar value is illegible; we recommend that it be removed from the figure and explicitly indicated in the caption.

## Author Response

Point by Point Response We thank the reviewers and editor for their thoughtful and constructive feedback. We have revised the manuscript extensively to address all concerns raised. In general, we have clarified the interpretation of antibody selectivity, strengthened the experimental support through additional controls, improved the statistical analysis, and expanded the methodological descriptions. Below we summarize the major changes and how they address the reviewers' concerns.

A central issue raised by both reviewers was the interpretation of antibody selectivity. We agree that the original terminology overstated the degree of conformational specificity, particularly for the trans antibody. Accordingly, we have revised the terminology throughout the manuscript from "conformation-specific" to "conformation-preferring". We now explicitly state that the cis antibody exhibits a stronger conformational preference, whereas the trans antibody behaves primarily as a phosphorylation-dependent antibody with limited conformational selectivity. The interpretation of Figure 1B has been updated to reflect this more nuanced view, and corresponding changes have been made in the Results and Discussion.

Both reviewers also raised concerns regarding whether the antibodies detect phosphorylation at T19 or the conformational state of the adjacent proline bond. To address this, we have added additional controls and clarified the interpretation of our data. Specifically, we have incorporated non-phosphorylated peptide controls in competition assays (Supplementary Figure 1) and performed peptide competition experiments using phosphorylated peptides. These results demonstrate that antibody recognition is phosphorylation-dependent, with conformational preference modulating binding rather than absolute conformational specificity. This interpretation has now been clearly stated throughout the manuscript.

Another major concern was the use of a high (10 mM) peptide concentration in competition assays, which could obscure differences in binding affinity. In response, we have performed competition titration experiments, including dose-response analyses under both wild-type and T19A conditions, and have estimated EC50 values. These data are now included in Figure 1 and Supplementary Figure 1 which provide a more quantitative assessment of antibody binding properties.

The reviewers also requested additional controls to establish antibody specificity. We have addressed this by adding multiple new controls, including a non-phosphorylated T19 peptide in competition assays (Supplementary Figure 1), a PSD-95 T19A mutant to assess phospho-specificity (Supplementary Figure 2), and untransfected HEK293T cells to establish baseline signal (Figure 2). Together with the peptide competition and phosphatase experiments, these data provide strong evidence that antibody recognition is phosphorylation-dependent.

Concerns were also raised regarding statistical power in imaging experiments. To address this, we have substantially increased the number of cells analyzed, ensured more balanced sampling across experimental conditions, and improved statistical analysis and reporting. Updated data are presented in Figure 1 and Supplementary Figure 1.

In addition, we have revised the overall interpretation of antibody function to avoid overstatement. We now consistently describe these reagents as phosphorylation-dependent, conformation-preferring reporters rather than strictly conformation-specific tools. This revised framing is reflected in the Abstract, Results, and Discussion.

We have also improved figure clarity and data presentation. Specifically, we modified Figure 1C, added Supplementary Figure 1B, moved selected panels from Figure 1 to the supplementary figures, and clarified labeling and quantification throughout. These changes improve the overall presentation and better support the conclusions.

To address concerns regarding peptide usage and purification steps, we have revised Table 1 to explicitly indicate the experimental step at which each peptide was used, along with the corresponding sequences and conditions. This clarification aligns the table with the experimental workflow.

Finally, we have expanded the Materials and Methods section to include a dedicated imaging (immunocytochemistry) subsection, along with additional details regarding experimental procedures and antibody usage.

In summary, the revised manuscript includes new specificity controls (T19A mutant, non-phosphorylated peptide, and untransfected cells), quantitative binding characterization through titration experiments, increased statistical rigor, improved figure organization and supplementary data, and a refined interpretation of antibody function. We believe these revisions fully address the concerns raised and significantly strengthen the manuscript.
