# Supplementary figures and images for: Development and Characterization of Conformation-Preferring Antibodies Targeting Phosphorylated Threonine 19 in PSD-95
Source: eNeuro. 2026 Jul 2;13(7):ENEURO.0016-26.2026. doi: 10.1523/ENEURO.0016-26.2026 (PMC13341226; doi:10.1523/ENEURO.0016-26.2026)

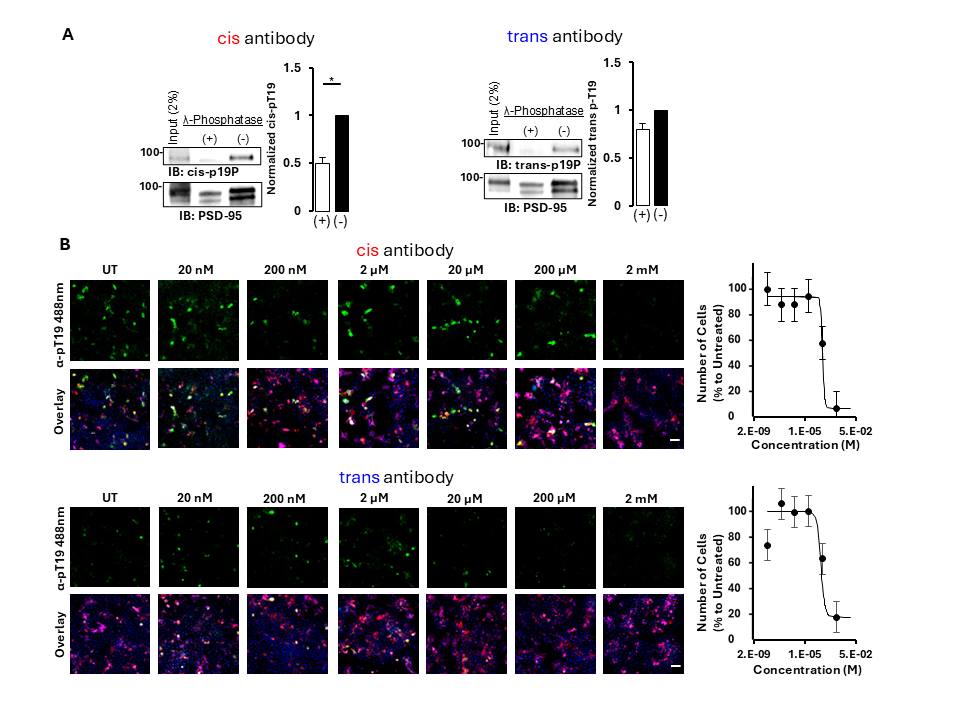

Supplement: Figure 1-1 — Antibodies demonstrate selectivity towards the phosphorylated versions of T19. (A) Western blot analysis showing phosphatase sensitivity and preference of the cis-pT19P and trans-pT19P antibodies. Treatment with λ-phosphatase resulted in decreased levels of phosphorylation detected by both the cis-pT19 (left) and trans-pT19 (right) antibodies. (B) Immunofluorescence imaging of pT19 at varying doses of non-phosphorylated peptide. HEK293 T cells co-expressing PSD-95 and HA-GSK3β labeled with conformation-specific antibodies, anti-PSD-95, and anti-HA. Scale bar: 50 µM. Quantification of cell number per field of view normalized to untreated condition (UT) and fitted to a sigmoidal dose-response curve presented to the right. Download Figure 1-1, TIF file. [file eneuro-13-ENEURO.0016-26.2026-s002.tif]

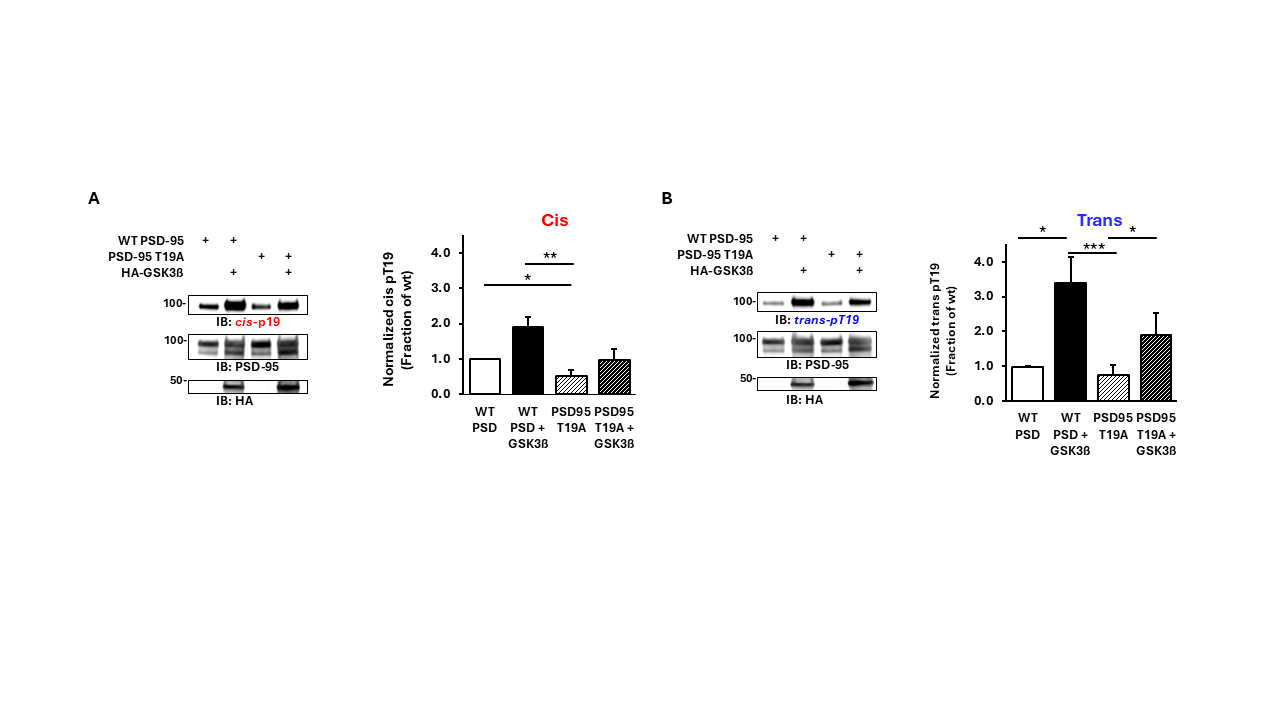

Supplement: Figure 2-1 — The cis-pT19 antibody displays higher preference towards the phosphorylatable form of PSD-95 at T19. Western blot images generated using the cis-pT19 (A) and trans-pT19 (B) antibodies from samples transfected with WT PSD-95 or PSD-95 T19A and HA-GSK3β. Graphs show quantification of normalized pT19 phosphorylation as a fraction of total PSD-95. Error bars represent SEM. Download Figure 2-1, TIF file. [file eneuro-13-ENEURO.0016-26.2026-s003.tif]
